# Correction: National assessment on the frequency of pain medication prescribed for intrauterine device insertion procedures within the Veterans Affairs Health Care System

**DOI:** 10.1371/journal.pone.0326620

**Published:** 2025-06-17

**Authors:** Anna D. Ware, Terri L. Blumke, Peter J. Hoover, Zach P. Veigulis, Jacqueline M. Ferguson, Malvika Pillai, Thomas F. Osborne

The Acknowledgments section for this paper is incorrect. The correct Acknowledgments section is as follows: Disclaimer: The findings and conclusions in this report are those of the authors and do not necessarily represent the views of the U.S. Department of Veterans Affairs or the United States Government. Additionally, Zach Veigulis and Thomas F. Osborne participated in the assessment during their employment with the U.S. Department of Veterans Affairs, but they are no longer affiliated with the VA.
