# Supplementary material for: National assessment on the frequency of pain medication prescribed for intrauterine device insertion procedures within the Veterans Affairs Health Care System
Source: PLoS One. 2025 Jan 10;20(1):e0308427. doi: 10.1371/journal.pone.0308427 (PMC11723627; doi:10.1371/journal.pone.0308427)
Supplement: S1 Appendix — (DOCX) [file pone.0308427.s001.docx]

| **Table A.1:** Individual Medications included in each medication group | |
| --- | --- |
| **Medication Group** | **Individual Medications** |
| **None** | None |
| **NSAIDs^1^** | Ibuprofen |
|  | Ketorolac |
|  | Naproxen |
| **Opioid Analgesics** | Hydromorphone |
|  | Fentanyl |
|  | Acetaminophen/Hydrocodone |
|  | Oxycodone |
|  | Tramadol |
|  | Codeine |
|  | Acetaminophen/Oxycodone |
|  | Buprenorphine/Naloxone |
|  | Acetaminophen/codeine |
|  | Meperidine |
|  | Morphine |
| **Prostaglandins** | Misoprostol |
|  | Dinoprostone |
| **Lidocaine** | Diclofenac (plus Cervical Lidocaine Gel) |
|  | Cervical Lidocaine Gel |
|  | Cervical Lidocaine Spray |
|  | Cervical Lidocaine-Prilocaine (LP) Cream |
|  | Paracervical Block |
| **Combination or Other** | Any combination of medications from two or more of the previous groups (i.e., misoprostol with ibuprofen). |

*^1^NSAIDs = Non-Steroidal Anti-inflammatory Drugs*
