# Supplementary figures and images for: National assessment on the frequency of pain medication prescribed for intrauterine device insertion procedures within the Veterans Affairs Health Care System
Source: PLoS One. 2025 Jan 10;20(1):e0308427. doi: 10.1371/journal.pone.0308427 (PMC11723627; doi:10.1371/journal.pone.0308427)

**Supplement 2**: Intrauterine Device Insertion Procedure Flowchart


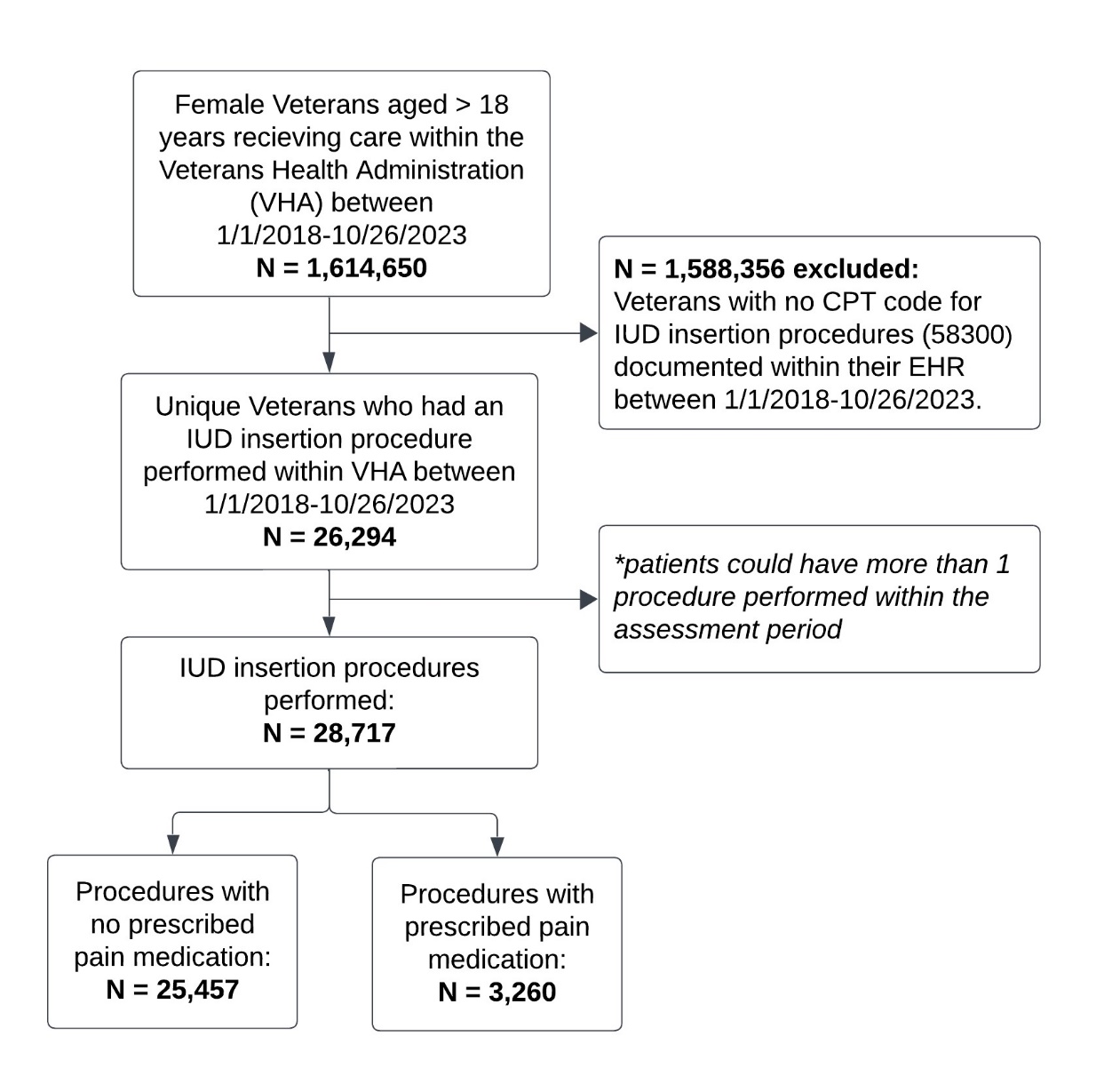

Supplement: S2 Appendix — (DOCX) [file pone.0308427.s002.docx]
