# Supplementary material for: National assessment on the frequency of pain medication prescribed for intrauterine device insertion procedures within the Veterans Affairs Health Care System
Source: PLoS One. 2025 Jan 10;20(1):e0308427. doi: 10.1371/journal.pone.0308427 (PMC11723627; doi:10.1371/journal.pone.0308427)
Supplement: S3 Appendix — (DOCX) [file pone.0308427.s003.docx]

**Table A.3**: Patient characteristics among the 24,010 IUD insertions performed within VHA between January 1st, 2018, to October 13th, 2023, with documented pain medication for their intrauterine device insertion procedure by medication type compared to those without any prescribed pain medication.

|  | **Total**  **(Column frequencies)** | **No Pain Medication** **Prescribed** | **NSAIDs** | **Combination or Other ^a^** | **Opioid Analgesics** | **Prostaglandins** | **Lidocaine ^e^** | ***P-value ^a^*** |
| --- | --- | --- | --- | --- | --- | --- | --- | --- |
| **Total, n (%)** | **28,717 (100.0%)** | **25,457 (88.7%)** | **2,392 (8.3%)** | **166 (0.6%)** | **171 (0.6%)** | **469 (1.6%)** | **62 (0.2%)** |  |
| ***Sociodemographic characteristics*** | | | | | | | |  |
| **Age (years), mean (SD)** | 39.53 (7.6) | 39.58 (7.6) | 39.06 (7.6) | 39.75 (7.3) | 39.52 (8.5) | 38.85 (8.2) | 38.74 (7.3) | 0.071 |
| **Race and/or Ethnicity, n (%)** |  |  |  |  |  |  |  | **<0.001** |
| American Indian or Alaskan Native | 412 (1.4%) | 378 (91.8%) | 26 (6.3%) | 1 (0.2%) | 1 (0.2%) | 5 (1.2%) | 1 (0.2%) |  |
| Asian | 884 (3.1%) | 773 (87.4%) | 74 (8.4%) | 7 (0.8%) | 7 (0.8%) | 20 (2.3%) | 3 (0.3%) |  |
| Black or African American | 7,165 (25.0%) | 6,246 (87.2%) | 728 (10.2%) | 46 (0.6%) | 23 (0.3%) | 107 (1.5%) | 15 (0.2%) |  |
| Hispanic | 3,951 (13.8%) | 3,513 (88.9%) | 329 (8.3%) | 21 (0.5%) | 22 (0.6%) | 57 (1.4%) | 9 (0.2%) |  |
| Native Hawaiian or Pacific Islander | 340 (1.2%) | 295 (86.8%) | 41 (12.1%) | 0 (0.0%) | 0 (0.0%) | 3 (0.9%) | 1 (0.3%) |  |
| Non-Hispanic White | 14,089 (49.1%) | 12,598 (89.4%) | 1,034 (7.3%) | 74 (0.5%) | 107 (0.8%) | 246 (1.8%) | 30 (0.2%) |  |
| Declined to Answer or Unknown | 1,876 (6.5%) | 1,654 (88.2%) | 160 (8.5%) | 17 (0.9%) | 11 (0.6%) | 31 (1.7%) | 3 (0.2%) |  |
| **Marital Status, n (%)** |  |  |  |  |  |  |  | **<0.001** |
| Married | 10,649 (37.1%) | 9,574 (89.9%) | 809 (7.6%) | 43 (0.4%) | 57 (0.5%) | 146 (1.4%) | 20 (0.2%) |  |
| Single | 8,620 (30.0%) | 7,427 (86.2%) | 847 (9.8%) | 72 (0.8%) | 56 (0.7%) | 197 (2.3%) | 21 (0.2%) |  |
| Divorced/Separated | 8,780 (30.6%) | 7,861 (89.5%) | 683 (7.8%) | 48 (0.6%) | 51 (0.6%) | 118 (1.3%) | 19 (0.2%) |  |
| Widowed | 143 (0.5%) | 127 (88.8%) | 13 (9.1%) | 0 (0.0%) | 2 (1.4%) | 1 (0.7%) | 0 (0.0%) |  |
| Unknown | 525 (1.8%) | 468 (89.1%) | 40 (7.6%) | 3 (0.6%) | 5 (1.0%) | 7 (1.3%) | 2 (0.4%) |  |
| **Has third party (non-VA) insurance, n (%)** |  |  |  |  |  |  |  | 0.329 |
| No | 19,351 (67.4%) | 17,153 (88.6%) | 1,634 (8.4%) | 106 (0.6%) | 112 (0.6%) | 311 (1.6%) | 35 (0.2%) |  |
| Yes | 9,348 (32.6%) | 8,286 (88.6%) | 758 (8.1%) | 60 (0.6%) | 59 (0.6%) | 158 (1.7%) | 27 (0.3%) |  |
| Unknown | 18 (0.1%) | 18 (0.0%) | 0 (0.0%) | 0 (0.0%) | 0 (0.0%) | 0 (0.0%) | 0 (0.0%) |  |
| **Rurality of Patient's Residence, n (%)** |  |  |  |  |  |  |  | **0.043** |
| Urban | 22,374 (77.9%) | 12,288 (88.4%) | 1,195 (8.6%) | 80 (0.6%) | 86 (0.6%) | 221 (1.6%) | 30 (0.2%) |  |
| Rural | 6,171 (21.5%) | 3,440 (90.7%) | 258 (6.8%) | 21 (0.6%) | 20 (0.5%) | 45 (1.2%) | 7 (0.2%) |  |
| Highly Rural | 133 (0.5%) | 72 (86.8%) | 9 (10.8%) | 0 (0.0%) | 0 (0.0%) | 2 (2.4%) | 0 (0.0%) |  |
| Unknown | 39 (0.1%) | 9,657 (88.3%) | 930 (8.5%) | 65 (0.6%) | 65 (0.6%) | 201 (1.8%) | 25 (0.2%) |  |
| ***Clinical characteristics*** | | | | | | | |  |
| **Body Mass Index, mean (SD)** | 31.30 (6.44) | 31.33 (8.97) | 31.41 (8.54) | 31.26 (7.15) | 29.42 (5.7) | 30.24 (6.85) | 30.39 (7.2) | 0.989 |
| **Parity Status *^b^*, n (%)** |  |  |  |  |  |  |  | **0.009** |
| Nulliparous | 27,114 (94.4%) | 23,997 (88.5%) | 2,277 (8.4%) | 162 (0.6%) | 162 (0.6%) | 457 (1.7%) | 59 (0.2%) |  |
| Parous/Multiparous | 1,603 (5.6%) | 1,460 (91.1%) | 115 (7.2%) | 4 (0.3%) | 4 (0.25%) | 12 (0.8%) | 3 (0.2%) |  |
| **Charlson Comorbidity Index, mean (SD)** | 0.57 (1.12) | 0.58 (1.13) | 0.51 (0.99) | 0.68 (1.04) | 0.63 (1.20) | 0.64 (1.21) | 0.63 (1.61) | 0.064 |
| ***Diagnosed Conditions*** |  |  |  |  |  |  |  |  |
| **Chronic pelvic pain, n (%)** |  |  |  |  |  |  |  | **<0.001** |
| Yes | 6,485 (22.6%) | 5,583 (86.1%) | 648 (10.0%) | 69 (1.1%) | 57 (0.9%) | 111 (1.7%) | 17 (0.3%) |  |
| No | 22,232 (77.4%) | 19,874 (89.4%) | 1,744 (7.8%) | 97 (0.4%) | 114 (0.5%) | 358 (1.6%) | 45 (0.2%) |  |
| **Dyspareunia, n (%)** |  |  |  |  |  |  |  | **<0.001** |
| Yes | 2,539 (8.8%) | 2,196 (86.5%) | 241 (9.5%) | 20 (0.8%) | 28 (1.1%) | 44 (1.7%) | 10 (0.4%) |  |
| No | 26,178 (91.2%) | 23,261 (88.9%) | 2,151 (8.2%) | 146 (0.6%) | 143 (0.6%) | 425 (1.6%) | 52 (0.2) |  |
| **Post-menopausal, n (%)** |  |  |  |  |  |  |  | 0.074 |
| Yes | 2,425 (8.4%) | 2,150 (88.7%) | 183 (7.6%) | 14 (0.6%) | 23 (1.0%) | 49 (2.0%) | 6 (0.3%) |  |
| No | 26,292 (91.6%) | 23,307 (88.7%) | 2,209 (8.4%) | 152 (0.6%) | 148 (0.6%) | 420 (1.6%) | 56 (0.2%) |  |
| **Dysmenorrhea, n (%)** |  |  |  |  |  |  |  | **<0.001** |
| Yes | 5,332 (18.6%) | 4,580 (85.9%) | 530 (9.9%) | 55 (1.0%) | 47 (0.9%) | 111 (2.1%) | 9 (0.2%) |  |
| No | 23,385 (81.4%) | 20,877 (89.3%) | 1,862 (8.0%) | 111 (0.5%) | 124 (0.5%) | 358 (1.5%) | 53 (0.2%) |  |
| **Anxiety Disorders *^c^*, n (%)** |  |  |  |  |  |  |  | **<0.001** |
| Yes | 18,004 (62.7%) | 15,896 (88.3%) | 1,514 (8.4%) | 125 (0.7%) | 129 (0.7%) | 304 (1.7%) | 36 (0.2%) |  |
| No | 10,713 (37.3%) | 9,561 (89.3%) | 878 (8.2%) | 41 (0.4%) | 42 (0.4%) | 165 (1.5%) | 26 (0.2%) |  |
| **Postpartum depression, n (%)** |  |  |  |  |  |  |  | 0.146 |
| Yes | 273 (1.0%) | 244 (89.4%) | 25 (9.2%) | 0 (0.0%) | 1 (0.4%) | 1 (0.4%) | 2 (0.7%) |  |
| No | 28,444 (99.0%) | 25,213 (88.6%) | 2,367 (8.3%) | 166 (0.6%) | 170 (0.6%) | 468 (1.7%) | 60 (0.2%) |  |
| **History of Military Sexual Trauma, n (%)** |  |  |  |  |  |  |  | **0.027** |
| Yes | 10,981 (38.2%) | 9,681 (88.2%) | 957 (8.7%) | 67 (0.6%) | 74 (0.7%) | 169 (1.5%) | 74 (0.7%) |  |
| No | 17,736 (61.8%) | 15,776 (89.0%) | 1,435 (8.1%) | 99 (0.6%) | 97 (0.6%) | 300 (1.7%) | 97 (0.6%) |  |
| ***^a^*** Statistical significance was determined by Chi-Square or Analysis of Variance (ANOVA) tests. Significant values (*p-value* < 0.05) are bolded.  ***^b^*** Data for this variable may include instances of missingness that could not be fully accounted for in the analysis  ***^c^*** Anxiety disorders included panic disorder (episodic paroxysmal anxiety), generalized anxiety disorder, other specific anxiety disorders, anxiety disorder (unspecified), and other mixed anxiety disorders. | | | | | | | |  |
