# Supplementary material for: National assessment on the frequency of pain medication prescribed for intrauterine device insertion procedures within the Veterans Affairs Health Care System
Source: PLoS One. 2025 Jan 10;20(1):e0308427. doi: 10.1371/journal.pone.0308427 (PMC11723627; doi:10.1371/journal.pone.0308427)
Supplement: S4 Appendix — (DOCX) [file pone.0308427.s004.docx]

**Table A.4**: Proportion of United States Women Veterans Prescribed Pain Medication for IUD Procedures by State, 2018-2023

| US State | Total Number of Procedures Performed, N | Proportion of procedures performed, % | Proportion where any pain medication was prescribed, % | Proportion where non-ibuprofen pain medication was prescribed, % |
| --- | --- | --- | --- | --- |
| New Hampshire | 122 | 0.4% | 32.0% | 10.7% |
| Puerto Rico | 169 | 0.6% | 30.2% | 5.3% |
| District Of Columbia | 424 | 1.5% | 28.1% | 26.7% |
| Maryland | 306 | 1.1% | 24.5% | 7.5% |
| Utah | 277 | 1.0% | 24.2% | 1.8% |
| Washington | 837 | 2.9% | 22.3% | 2.4% |
| South Carolina | 600 | 2.1% | 20.2% | 3.7% |
| Wyoming | 76 | 0.3% | 19.7% | 6.6% |
| Michigan | 556 | 1.9% | 18.7% | 12.9% |
| Ohio | 801 | 2.8% | 17.6% | 3.5% |
| Florida | 2993 | 10.4% | 16.9% | 9.3% |
| Oklahoma | 372 | 1.3% | 16.7% | 5.4% |
| Arkansas | 217 | 0.8% | 15.7% | 14.7% |
| Massachusetts | 245 | 0.9% | 15.5% | 5.7% |
| Idaho | 227 | 0.8% | 15.4% | 14.1% |
| Tennessee | 759 | 2.6% | 14.6% | 2.1% |
| Arizona | 910 | 3.2% | 13.4% | 4.9% |
| Rhode Island | 147 | 0.5% | 12.9% | 8.8% |
| South Dakota | 135 | 0.5% | 12.6% | 4.4% |
| Louisiana | 315 | 1.1% | 12.4% | 7.6% |
| New Mexico | 225 | 0.8% | 11.6% | 2.7% |
| Alabama | 281 | 1.0% | 11.0% | 10.7% |
| Illinois | 1463 | 5.1% | 11.0% | 7.0% |
| California | 2827 | 9.8% | 10.8% | 4.4% |
| New Jersey | 179 | 0.6% | 10.6% | 8.4% |
| Colorado | 829 | 2.9% | 10.0% | 9.5% |
| North Dakota | 82 | 0.3% | 9.8% | 8.5% |
| Iowa | 117 | 0.4% | 9.4% | 5.1% |
| North Carolina | 1402 | 4.9% | 9.3% | 2.2% |
| Maine | 98 | 0.3% | 9.2% | 7.1% |
| West Virginia | 228 | 0.8% | 9.2% | 3.1% |
| Indiana | 326 | 1.1% | 8.9% | 7.4% |
| Delaware | 80 | 0.3% | 8.8% | 1.2% |
| Wisconsin | 430 | 1.5% | 8.1% | 3.3% |
| Nebraska | 212 | 0.7% | 8.0% | 3.3% |
| Georgia | 861 | 3.0% | 7.5% | 3.7% |
| Pennsylvania | 938 | 3.3% | 6.8% | 4.2% |
| Hawaii | 91 | 0.3% | 6.6% | 3.3% |
| New York | 712 | 2.5% | 6.6% | 4.1% |
| Nevada | 508 | 1.8% | 6.3% | 4.3% |
| Minnesota | 497 | 1.7% | 6.0% | 2.0% |
| Texas | 3203 | 11.2% | 4.4% | 2.8% |
| Vermont | 74 | 0.3% | 4.1% | 1.4% |
| Kentucky | 326 | 1.1% | 4.0% | 1.8% |
| Virginia | 907 | 3.2% | 3.7% | 1.7% |
| Kansas | 138 | 0.5% | 3.6% | 2.9% |
| Mississippi | 92 | 0.3% | 3.3% | 2.2% |
| Oregon | 458 | 1.6% | 3.3% | 2.6% |
| Connecticut | 198 | 0.7% | 2.5% | 2.0% |
| Missouri | 315 | 1.1% | 1.0% | 0.6% |
| Cumulative Total | **28717** | **100.0%** |  |  |
