# Supplementary material for: National assessment on the frequency of pain medication prescribed for intrauterine device insertion procedures within the Veterans Affairs Health Care System
Source: PLoS One. 2025 Jan 10;20(1):e0308427. doi: 10.1371/journal.pone.0308427 (PMC11723627; doi:10.1371/journal.pone.0308427)
Supplement: Attachment — Submitted filename: Reviewer_Response.docx [file pone.0308427.s005.docx]

PLOS ONE

1265 Battery Street Suite 200

San Francisco, CA 94111

September 23, 2024

RE: Manuscript Number: PONE-D-24-29921

Dear Editors,

Thank you for the opportunity to revise our manuscript entitled, “National Assessment on the Frequency of Pain Medication Prescribed for Intrauterine Device Insertion Procedures within the Veterans Affairs Health Care System” for publication in *PLOS ONE*. We sincerely appreciate the reviewers’ feedback and have made considerable efforts to address all comments. Please see below the point-by-point response to the reviewers.

**Reviewer 1**: Thanks for the opportunity to review this manuscript. The topic is extremely important and addresses a gap in the literature surrounding healthcare for women Veterans in the form of IUD and associated pain management. Contraceptive care for women is understudied, pain during IUD insertion is understudied, and each of these topics among Veterans specifically is remarkably understudied. Thanks to the authors for contributing to this area of work!

**Response**: Thank you for your kind assessment of our work. We greatly appreciate your thoughtful and thorough suggestions as we aim to address this important knowledge gap in scientific literature.

**Reviewer 1**: Is there other relevant work regarding pain medications among Veterans, and in particular, disparities between men and women Veterans that would be helpful to report in the introduction? While the IUD is a procedure that occurs in individuals assigned female sex at birth, the disparity in pain management between male and female Veterans is relevant here.

**Response**: Thank you for your insightful comment. Recent literature among Veterans does suggest some differences in pain management between male and female Veterans, although the findings are nuanced. For instance, Driscoll et al. (2017; https://doi.org/10.1093/pm/pnx023) found no significant differences in pain management methodologies between male and female Veterans. However, Murphy et al. (2016; https://www.rehab.research.va.gov/jour/2016/531/pdf/jrrd-2014-10-0250.pdf) found that while women reported shorter pain duration and were more likely to experience head or limb pain, they were initially prescribed fewer opioids compared to men. Despite this, opioid use did not differ significantly at follow-up. Additionally, Oliva et al. (2015; https://doi.org/10.1111/pme.12501) noted that women Veterans prescribed pain medications were more likely to receive guideline-recommended practices than their male counterparts.

Further evidence of perceived bias among female Veterans indicates that gender-based assumptions may also influence care. For example, Mattocks et al. (2020; <https://doi.org/10.1016/j.whi.2019.10.003>) found that some female Veterans felt their symptoms were not taken seriously by VA specialty care providers, attributing this to gender-based assumptions, such as being told that their symptoms were due to hormonal fluctuations. These findings underscore that, while explicit disparities in pain management methodologies may not be significant, perceptions of gender bias in care remain an critical issue.

Given the complexity and sometimes conflicting results of these studies, expanding the introduction to cover gender-based differences in pain management may divert the paper’s focus. Instead, we have chosen to address the issue of perceived gender bias within our discussion section. To this end, we have added the following sentences:

“A 2020 VHA study further highlighted these perceptions, with some female Veterans reporting that VA specialty care providers did not take their symptoms seriously, attributing their health concerns to hormonal fluctuations. By initiating standardized and data informed clinical guidelines for providers to counsel patients on what to expect during IUD placement, including effective pain control options, we believe that VHA will not only provide better care, but also strengthen the trust between Veterans and their providers.”

Additionally, we have expanded the discussion to suggest that further exploration of pain management differences between male and female Veterans is warranted, as some research highlights potential variations in pain management methods and perceived biases. The added sentence is:

“Additionally, further exploration of pain management differences between male and female Veterans, as some research indicates variations in methods and perceived biases, could help address any gender-related disparities in care.”

In this expanded section of the discussion, we reference both Mattocks et al. (2020) and Olivia et al. (2015) to emphasize the importance of addressing potential biases and ensuring equitable pain management for all Veterans.

**Reviewer 1**: The authors might give a bit more attention to what effective pain management strategies is and are not in the introduction for context that would help to interpret later findings.

**Response:** Thank you for your suggestion. We agree that being more specific on which pharmaceuticals are effective in the introduction could be help with the interpretation of our later findings. We have added to the introduction to now state:

“In attempts to mitigate pain during IUD insertion, several pharmacological interventions have been tested with mixed results. For example, certain formulations of lidocaine, such as cervical lidocaine gel and paracervical blocks, have shown effectiveness in reducing pain during the procedure. In contrast, most non‐steroidal anti‐inflammatory drugs (NSAIDs), including ibuprofen and ketorolac, have generally been ineffective at managing insertion-related pain, while naproxen has demonstrated some promise. Cervical ripening agents (prostaglandins), like misoprostol, were once thought to ease the insertion process, but recent studies have also cast doubt on their effectiveness. Additionally, while opioids are not commonly prescribed for this procedure, tramadol has been shown to reduce pain during IUD insertion in some studies.”

**Reviewer 1**: Authors might note why IUD is preferable or the only option for some individuals when considering modality of contraception.

**Response:** Thank you for your comment. We agree that this could be a value add to our introduction. Therefore, we have expanded on the first paragraph of the introduction to now state:

“For some individuals, IUDs may be the preferable or only option for contraception due to specific health conditions. Those who cannot tolerate hormonal contraception, such as individuals with migraines, hypertension, or those at increased risk of thromboembolism, may opt for hormone-free copper IUDs. Additionally, hormonal IUDs also provide non-contraceptive benefits, such as reducing heavy menstrual bleeding and dysmenorrhea.”

**Reviewer 1**: The authors note that Veterans are more likely to have IUD than non-Veterans. Is there any work on why this difference exists? Is there evidence that VHA providers are more likely to advise IUD vs. oral contraception or other methods? This is particularly interesting considering the high rates of MST among women Veterans and research demonstrating that some individuals with sexual trauma may avoid internal exams, pap smears, or other aspects of gynecological care.

**Response:** Thank you for your comment. The study we reference by Koenig et al. (2019), entitled “Factors associated with long-acting reversible contraception use among women Veterans in the ECUUN study,” found that increased use of LARC was not linked to provider- or facility-level factors. However, it did find that women receiving care VA women's health clinics more likely to receive LARCs. The author’s note that this “may suggest that requisite training of VA primary care providers as women's health providers, if practicing within a women's health clinics, along with a high target of women Veterans being seen by these women’s health providers allows providers, regardless of their location and characteristics, to provide comprehensive care for women.” While there is no direct evidence showing that VHA providers recommend IUDs over oral contraception, the structure of VA care – offering no or minimal cost for all contraceptive options – coupled with the focus on comprehensive primary care, ensures that Veterans have easier access to LARCs. This may contribute to the higher rates of LARC use, including IUDs, among Veterans compared to the general population, where cost and access barriers are more prevalent. Additionally, LARCs are often preferred for their long-term efficacy and convenience, which could be a factor for Veterans seeking reliable and low-maintenance contraception.

To clarify this in our paper, we expanded the second to last paragraph of the introduction to now state:

“To meet the needs of this rapidly expanding population, VHA has focused on key initiatives to enhance access to comprehensive women’s healthcare through specifically trained providers. Veterans can now access a full range of contraceptive options with no or minimal out-of-pocket expenses as part of their comprehensive primary care. This enhanced accessibility, coupled with the availability of specifically trained providers in VA women’s health clinics, enables Veterans to access LARCs more readily than the general population. Consequently, LARC utilization rates, including IUDs and implants, have been reported to be higher among Veterans than in the general population. According to a national telephone survey, approximately 23% of Veterans assigned female at birth (AFAB) of childbearing age (18-45 years) at risk for unintended pregnancy utilize a LARC, compared to only 11% of the general US population. This survey also found that 65% of these Veterans received their IUD within a VA outpatient clinic.”

**Reviewer 1**: Are there other disparities to be mindful to acknowledge, such as racial or ethnic disparities, socioeconomic disparities? There is significant historical context around gynecological care for Black women, for example, that may impact treatment of pain as well as the actual physician recommendations.

**Response:** Thank you for your comment. Racial and socioeconomic disparities in gynecological care have been well documented. We have added a sentence to our discussion section to acknowledge this disparity:

“Racial and socioeconomic disparities in pain management, particularly in gynecological care, are well-documented, with historical inequities affecting the treatment of Black women. In our assessment, we found slightly higher proportions of Black (12.8%) and Native Hawaiian or Pacific Islander (13.2%) patients prescribed pain medication compared to Non-Hispanic Whites (10.6%) and American Indian or Alaskan Natives (8.3%).”

We also added the following reference: Hoffman KM, Trawalter S, Axt JR, Oliver MN. Racial bias in pain assessment and treatment recommendations, and false beliefs about biological differences between blacks and whites. Proc Natl Acad Sci U S A. 2016;113(16):4296-4301. doi:10.1073/pnas.1516047113

**Reviewer 1**: It’s likely beyond the scope of this study, but it would be interesting to comment on the impact of Roe v Wade being overturned as related to contraception recommendations (particularly in states where abortion is banned).

**Response:** Thank you for your insight regarding the impact of the overturning of Roe v. Wade on contraception recommendations. We agree that this is a critical issue and have added to the discussion acknowledging the potential influence of this legal change on contraceptive choices, particularly in states where abortion access is restricted or banned. While it is beyond the scope of our current assessment, we have highlighted that future research could explore how these shifts affect IUD use within the VA and nationally:

“Given the recent legislative changes surrounding reproductive rights, such as the overturning of Row v. Wade, there is increasing attention on the accessibility of contraceptives, particularly in states where abortion access is restricted or banned. This may influence both patient preferences and provider recommendations for IUDs as a highly effective and long-term method to prevent unintended pregnancies. Although our assessment does not specifically assess the impact of these legal changes, future research could explore how shifts in reproductive healthcare access affect the utilization of IUDs and other forms of contraception within the VHA and nationally.”

**Reviewer 1**: The early sentences of the Discussion section largely mirror the early sentences in the introduction; consider reframing discussion section around how your manuscript addresses gaps in the literature.

**Response:** Thank you for your comment. We have revised this section of the discussion to center more on how our manuscript addresses gaps in literature:

“This assessment provides new insights into the current state of prescribed pain management for Veterans undergoing IUD insertions within the VHA, addressing gaps in the literature regarding the adequacy of pain interventions in this population. Although pain associated with IUD insertion is well documented, our assessment revealed that only 11.4% of the 28,717 IUD insertions procedures performed at VHA, nationally, during the assessment period, had any form of documented pain medication, highlighting the opportunity to improve patient care.”

**Reviewer 1**: I might note that the “steady increase” in medications prescribed is relatively unimpressive given that IUD insertion pain is not new information. This is in no way meant as a critique of the authors, but a suggestion to make your statements stronger to pull for systemic change in guidance. The authors suggest reasons for variation in prescribing practices; I would also include bias.

**Response:** Thank you for your thoughtful comment. We agree that the wording of “steady increase” does not adequately reflect the fact that it remains a very slow adoption rate of pain interventions offered for this procedure. We have revised this section to now state:

“Despite an average annual increase of 0.52% in the proportion of those prescribed pain management between January 1st, 2018, to October 13th, 2023, this slow progression in the adoption of pain management strategies highlights the need for more systemic change in clinical guidance to ensure that effective pain relief is consistently offered.”

Additionally, we have added a sentence to also suggest potential bias as a reason for variation in prescribing practices:

“While our data could not assess the potential causes of the variation, possible factors include regional practices, provider training, patient preferences, and/or specific protocols within each VHA facility. The absence of a standardized operating procedure or mandate for pain management may contribute to these inconsistencies. Bias, including both unconscious bias and gender-related biases, may also play a role in prescribing practices, as previous research has shown that women’s pain is often under-treated in healthcare settings.”

**Reviewer 1**: It looks like between-group differences for MST, rurality, anxiety disorders, and chronic pelvic pain were examined but only noted in the table. It may be worth pointing readers to this in the text, as these are interesting findings that, without close review of the table, may be missed.

**Response:** Thank you for your insight. We have added a sentence to the results section to discuss the rurality of patient’s resident results:

“Additionally, 11.9% of patients with residing in an urban location were prescribed pain medication compared to only 9.4% of patients residing in a rural location (p=0.003; Table 1).”

We also added a sentence to the discussion to direct the readers to these findings:

“Notably, we observed significant between-group differences for several patient characteristics. Patients with a history of MST, anxiety disorders, dysmenorrhea, dyspareunia, or chronic pelvic pain were more likely to be prescribed pain medication than those without these conditions. Similarly, we found differences based on rurality, with 11.9% of urban patients receiving pain medication compared to 9.4% of rural patients.”

**Reviewer 1**: In the recommendations, it may be important to emphasize a top-down recommendation or requirement that providers have these medication discussions prior to the procedure. Similar to how VHA providers are trained in other procedures (i.e., empirically supported therapy rollouts), it seems urgent that providers are told, not asked, what to do to better align with best practice.

**Response:** Thank you for your comment. We agree that emphasizing a top-down recommendation, as seen with other procedures within VHA, is important. We have added to the discussion to now state:

“Our findings allow for data-informed development and adoption of evidence-based guidelines and the standardization of pain management protocols within the VHA. Enhancing provider education is paramount to ensure healthcare professionals are well-informed about effective pain control options. It is essential that top-down systemic recommendations or requirements, mandated by leadership, be implemented to ensure that providers consistently discuss pain management options with patients prior to IUD insertion procedures. Similarly to how the VHA has successfully rolled out training for other procedures, such as empirically supported therapies, these organization-wide policies would standardize care and improve alignment with evidence-based practices. Pre-procedural discussions would ensure that patients are informed of their pain management options and reduce disparities in treatment. Additionally, promoting patient-centered care models that respects the unique needs of Veterans who are AFAB and integrating patient feedback into care processes will be essential. These measures will ensure that pain management during IUD insertion procedures is both effective and tailored to individual patient needs.”

We also added the following reference: Karlin BE, Cross G. From the laboratory to the therapy room: National dissemination and implementation of evidence-based psychotherapies in the U.S. Department of Veterans Affairs Health Care System. Am Psychol. 2014;69(1):19-33. doi:10.1037/a0033888

**Reviewer 1**: Did the authors distinguish between pain medications prescribed within 1 day of the insertion procedure and those within 44 days? This may help speak to the last issue mentioned in the limitations section, where authors note that these rates may be underestimates of the true rates.

**Response**: Thank you for your insightful comment. While distinguishing between pain medications prescribed within 1 day of the procedure and those prescribed within the 44 days prior could theoretically provide additional clarity, it would be challenging to accurately separate these cohorts due to the nature of how medications are ordered and used in clinical practice. In many cases, providers may order pain medications during the consult visit for the patient to pick up in preparation for the procedure, but those medications may not actually be used until the day of the procedure. Additionally, we found that quite a few orders were written on the day of the procedure, likely as part of a protocol or standard order for the procedure itself.

Unfortunately, without a reliable method to measure compliance with prescribed medications, it would be difficult to determine whether medications prescribed earlier were used at the time of the procedure. Given these limitations, we opted for a comprehensive 45-day window to capture any potential pain medications revealed to the IUD procedure. While this approach increases sensitivity, it may overestimate the number of medications intended for IUD-associated pain.

**Reviewer 1**: Future steps may include qualitative interviews with key stakeholders, including Veterans, providers, administrators, etc., to better understand the choices, as well as the patient-provider communications that occur surrounding the choice to use an IUD and the associated pain.

**Response:** Thank you for your comment. We agree that this would be a crucial value add to future work and have expanded this section of the discussion to now state:

“Future steps could include qualitative interviews with key stakeholders (such as Veterans, providers, and administrators) to better understand the decision-making processes surrounding the use of IUDs, as well as the patient-provider communications that occur regarding pain management. Understanding the perspectives of these stakeholders will be crucial in developing strategies that align with both patient needs and provider capabilities.”

**Reviewer 2**: This study examined frequency and type of pain medication treatment for intrauterine device (IUD) insertion procedures within the Veterans Health Administration. The authors reported that 11.4 percent of procedures had any form of prescribed pain medication identified, with ibuprofen being the most common type of pain medication even though it is not effective. This well-done study is novel in its use of large electronic health records (EHR) data to assess prevalence of pain medication treatment at the population level, trends in pain medication treatment over time, and differences in pain medication treatment by patient, facility, and provider characteristics.

**Response:** Thank you for the kind and thoughtful assessment of our work.

**Reviewer 2**: The authors need to describe more carefully and critically evidence regarding pain among people receiving IUDs, including how pain is measured. Their main evidence is a study according to which 78 percent of respondents reported moderate to severe pain levels, but that study only had 109 participants and focused on a specific population (nulliparous women aged 18-30; see reference number 5). Another study they mentioned (reference number 3) seemed to find a much lower level of reported pain (a median level of pain of 1.0cm on a 10cm visual analog scale), whereas a third study (reference number 47) reported a mean patient maximum pain of 6.48cm on the same scale. Moreover, studies on the effectiveness of pain medication report pain levels for the control group and thus provide further evidence (see for instance Table 2 in https://doi.org/10.1016/j.contraception.2011.10.015; Table 2 in https://doi.org/10.1016/j.contraception.2014.11.012; and Table I in https://doi.org/10.1016/j.ajog.2006.08.022). It seems as if the reported level of pain varies considerably across studies, which may be due to pain measurement, patient characteristics, or random sample variation. A more thorough description of reported pain among people receiving IUDs is important because the main take-away of this study was that levels of prescribed pain medication was low compared to the pain patients experienced.

Related to this point, it seems as the VA EHR data do not include reported pain of patients as part of IUD insertion procedures. This type of information would be very helpful to be able to assess overall pain levels in this population, and to examine disparities between experienced pain and pain medication treatment. The authors should clarify this aspect as a limitation of their study. They could also consider adding routine collection of pain levels as a recommendation.

**Response:** Thank you for your insightful comment. We agree that it is important to highlight the variability in patient-reported pain levels during IUD insertion, as different studies report a wide range of findings. To address this, we added the following to the discussion’s limitation section:

“It is important to note that evidence regarding pain levels among individuals receiving IUDs varies considerably across studies, likely due to differences in pain measurement methods, patient characteristics, and sample sizes. Some studies report moderate to severe pain, with mean scores of 64.8 mm on a 100 mm VAS, while others show lower pain levels, with mean scores ranging from 1.0 cm on a 10 cm VAS to 38.0 mm on a 100 mm VAS during insertion. This variability underscores the need for consistent pain assessment methods and highlights that pain perception can differ significantly across individuals.”

Additionally, we have revised the introduction to better reflect the variability in pain experiences:

“Although patient-reported pain for this procedure can vary, a recent survey reported that 78% of respondents rated their IUD insertion pain as moderate to severe, with 46% experiencing vasovagal symptoms.”

We have also added references to the newly published CDC clinical guidelines, which emphasize the importance of discussing pain management options with patients prior to IUD insertion:

- Introduction: “Notably, the Center for Disease Control and Prevention (CDC) updated its guidelines in 2024 asking providers to discuss effective pain management strategies with their patients prior to IUD placement.”
- Discussion: “Similarly, in 2024, the CDC updated their “US Selected Practice Recommendation for Contraceptive Use” following a review of scientific evidence and consultation with national experts. These updated recommendations include guidance on the provision of medication for IUD placement, as well as management of bleeding irregularities during implant use.”

Regarding the VA EHR data, we acknowledge that the absence of patient-reported pain scores is a limitation of our assessment. To address this, we have clarified in the limitations section that pain was not consistently documented for most procedures. Specifically, there was 74% missingness in pre-procedure scores and 99% missingness in pain scores recorded during or after-insertion). For those patients with documented scores, the method of documentation did not allow us to determine at what point the pain score was recorded, which is critical for assessing pain experienced at the time of insertion. As a result, we could not directly analyze the relationship between reported pain and pain management interventions. To mitigate this limitation in future studies, we recommend integrating routine collection of pain levels at the time of IUD insertion into clinical practice. This has been included in the limitations and future steps section:

“Unfortunately, we were unable to capture patient-reported pain scores for the majority of IUD insertions in this assessment, as pain was not consistently documented before, during, or after the procedure. Additionally, for the few with documented scores, due to the methods of documentation, we were unable to ascertain at what time the pain score was recorded, which is critical when assessing perceived pain at the time of insertion. This limitation prevented us from directly analyzing the relationship between reported pain and the use of pain management interventions. Given the importance of understanding patients’ pain experiences during this procedure, we recommend that routine collection of pain levels at the time of IUD insertion be integrated into clinical practice.”

“…future work could incorporate the routine collection and analysis of patient-reported pain scores during IUD insertion. This would allow for a more direct assessment of pain levels in relation to prescribed interventions and provide valuable insights into optimizing pain management strategies for our specific patient population.”

**Reviewer 2**: Materials and Methods. It was not clear to me why the authors included some of the covariates, especially those related to facilities (e.g., facility complexity). The authors should clarify their motivation for including these covariates. They should also add this aspect of their analysis in the last paragraph summarizing their study.

**Response:** Thank you for addressing this issue. We have added clarification on why we chose to include our covariates to our methods section:

“We included a range of patient sociodemographic and clinical characteristics to account for potential disparities in care and factors that may influence access to care. Sociodemographic variables such as age, self-identified race or ethnicity, marital status, third-party (non-VA) insurance, and rurality of patient’s residence were included to capture differences in healthcare access and utilization. In addition, clinical characteristics like body mass index (BMI), parity status (nulliparous vs. parous), Charlson Comorbidity Index (CCI) score, and female-specific comorbidities (e.g., chronic pelvic pain, postpartum depression, dyspareunia, history of military sexual trauma (MST)) were included to identify factors that may influence an individual’s medical need for pain management.

We also captured provider and facility characteristics including the 4 US regions (as defined by the Centers for Disease Control, 2023), facility complexity (VHA classification system for VA medical centers that ranges from high, medium, and low) , state location of the clinic, outpatient care setting (e.g., gynecology clinic, primary care clinic), and the type of primary provider (e.g., gynecologist, nurse practitioner) associated with the IUD insertion procedure. Facility complexity was included to account for differences in resources availability, which may influence pain management practices. High complexity facilities have larger levels of patient volume, higher patient risk, more teaching and research resources, and contain level 4 to 5 intensive care units compared to low complexity facilities which have little or no teaching/research, the lowest number of physician specialists per patient, and contain level 1 and 2 intensive care units.”

**Reviewer 2**: Results. I was surprised by the small fraction of IUD insertion procedures relative to the number of adults patients who were assigned female at birth (28,717 of 1,614,650, or 1.8 percent), given that 23 percent of Veterans assigned female at birth and ages 18-45 reported use of some long-active reversible contraception (see introduction, p.4). The authors should explain this discrepancy.

**Response:** Thank you for your comment. The 23% LARC use rate reported in the introduction is based on data from a national telephone survey that included 987 women Veterans at risk of unintended pregnancy. This rate reflects LARC use among those surveyed, which may include both IUDs and implants rather than just IUD insertions. In contrast, our assessment focuses solely on IUD insertions that were captured within VA EHR data among all Veterans assigned female at birth, nationally. This may explain the lower percentage observed in our study. We have revised the sentence in the introduction to clarify the reference’s study parameters to avoid confusion for the lower percentage later in the paper:

“Consequently, LARC utilization rates, including IUDs and implants, have been reported to be higher among Veterans than in the general population. According to a national telephone survey, approximately 23% of Veterans assigned female at birth (AFAB) of childbearing age (18-45 years) at risk for unintended pregnancy utilize a LARC, compared to only 11% of the general US population. This survey also found that 65% of these Veterans received their IUD within a VA outpatient clinic.”

**Reviewer 2**: Results. Table 1 shows prevalences of no pain prescription and *any* pain medication prescription by patient characteristics, whereas Table 2 shows prevalences of no pain prescription vs. *type* of pain prescription by facility and procedure characteristics. It would be helpful to harmonize these displays.

**Response:** Thank you for your suggestion. To provide more a granular depiction of these results, we have expanded upon Table 1, mirroring the structure of Table 2, and have included this as a supplemental table (Appendix 3). While disparities in the type of medications being prescribed to Veterans are noted within this supplemental table, considerations into the patient’s medical history and current needs must be made. As differences in how these medications may be prescribed, we chose to include this expanded table as supplemental information while discussing the overall findings and results within the main body of the manuscript.

Thank you for your time and consideration.

Sincerely,

Anna

Anna D Ware, MPH

Data Scientist

National Center for Collaborative Healthcare Innovation (NCCHI)

VA Palo Alto Health Care

VA Mobile: +1 (612) 369-2790
